# Letter to the Editor

**DOI:** 10.5005/jp-journals-10005-1539

**Published:** 2018-10-01

**Authors:** Saravana K Balasubramanian, Ishwarya Gurucharan, Mahalaxmi Sekar

**Affiliations:** 1 Reader, Department of Conservative Dentistry and Endodontics, SRM Dental College, SRM University, Ramapuram, Chennai, Tamil Nadu, India; 2 Postgraduate Student, Department of Conservative Dentistry and Endodontics, SRM Dental College, SRM University, Ramapuram, Chennai, Tamil Nadu, India; 3 Professor and Head, Department of Conservative Dentistry and Endodontics, SRM Dental College, SRM University, Ramapuram, Chennai, Tamil Nadu, India

**Keywords:** Cold, Pain, Precooling.

## Abstract

Pain is an important aspect of pediatric dentistry. Phlenophobia or fear for needle is one of the significant factor for reduced apprehensiveness and decreased pain threshold in pediatric patients.

**How to cite this article:** Balasubramanian SK, Gurucharan I, Sekar M. Letters to the Editor. Int J Clin Pediatr Dent, 2018;11(5):357-358

We read with great interest the article entitled “Efficacy of Different Precooling Agents and Topical Anesthetics on the Pain Perception during Intraoral Injection: A Comparative Clinical Study”, published in your esteemed journal [Int J Clin Ped Dent, May-August 2015; 8(2):119-12]. It was a good clinical study comparing the efficacy of the refrigerant (1, 1, 1, 3, 3-pentafluoropropane/1, 1, 1, 2-tetrafluoroethane), benzocaine and ice on the pain perception during intraoral injection using visual analog scale (VAS) and sound, eye, motor (SEM) scale. However, we would like to share express our thoughts views regarding a few observations in the current study.

The authors have employed custom-made ice cones (group IA) for precooling the injection site. However, there was no mention of the temperature of the ice cones, nor changes variations in their temperature from the preparation time, to their removal from the refrigerator and the actual time of application time. In addition, the contact time of ice cone in the present study was 1 minute which is in concomitant with that of a study conducted by Aminabadi et al.^1^ However, the clinical tolerance level of the oral tissues to a continuous cold exposure for 1 minute is highly questionable, particularly in pediatric cases, where adequate patient cooperation is a restraining factor. This is a clinically significant determinant since extreme cold exposure over a prolonged time induces localized constriction of blood vessels vasoconstriction, vasospasm, and transient peripheral vasodilation for arterio-venous communication. In addition to this, at temperatures below 2° C, tissues begin to freeze, and ice crystals start to form extracellularly within the interstitium when there is a drop in temperature less than below 2° C. This process results in a hyperosmolar state in the interstitium and plasma, subsequently imbibing drawing water from the intercellular network compartment, following which, cellular dehydration and mechanical disruption of cells by ice crystals occur.^1^ When rewarming begins, the reversed water influx leads to intracellular swelling and fluid leaks from the disrupted capillaries into the interstitial space, resulting in edema.^2^ In progressive injuries, further, an increase in prostaglandin F2 (alpha) and thromboxane levels results in clot formation, besides, platelet and leukocyte aggregation as a protective mechanism. Subsequently, arterial, capillary and venous thrombosis of blood vessels occur leading to an increase in swelling followed by tissue necrosis in response to progressive thrombosis.^2^ Hence, a detailed discussion an insightful discussion regarding the effects of cryoanesthesia would have been more appropriate. On the contrary, the contact time of the refrigerant (i.e., Gebauer’s Pain ease application in group IIA) employed was only 5 seconds which was not justified. The authors could have employed a similar contact time in group IA (i.e., ice cone application) for better appreciation and standardization of the study.

Also, the results of the present study revealed The current study also concluded that ice cone exhibited a significant increase in efficacy as compared to that of benzocaine gel and refrigerant. The reasons attributed to the increased efficiency of ice (site IA) were: (a) its increased contact time with the tissues as compared to the refrigerant (site IIA) and (b) this increased contact time of ice might have resulted in slowing the velocity of nerve impulse induction of almost all the types of nerve fibers. However this could have been explained further by highlighting the analgesic effects of cold therapy^3,4^ which are as follows: (i) Cold results in vasoconstriction of the blood vessels, thereby reducing the tissue metabolism and localized inflow of pain-inducing substances, decreasing edema formation. (ii) Reduction in temperature results in slower conduction of peripheral nerves, in addition to the fact that myelinated nerve fibers are more prone to this decreased conduction than unmyelinated nerve fibers. (iii) Topical application of cold Topical cold application also stimulates fast conducting A-delta nerve fibers myelinated Aδ fibers, activating inhibitory pain pathways, thereby which in turn raises the pain threshold. (iv) Cold also Cooling also results in reduced pain perception due to counterirritant effect by the thermal receptors and (v) Decreased neuromuscular transmission. Hence, the authors can consider further redefine the study by considering those mentioned above clinically relevant observations for a better appreciation of the results.
